# FOXC1, the new player in the cancer sandbox

**DOI:** 10.18632/oncotarget.22742

**Published:** 2017-11-28

**Authors:** Fahed A. Elian, Elizabeth Yan, Michael A. Walter

**Affiliations:** ^1^ Department of Medical Genetics, Faculty of Medicine and Dentistry, University of Alberta, Edmonton, Alberta, Canada

**Keywords:** transcription factor, breast cancer, basal-like

## Abstract

In recent years, rapidly accumulating evidence implicates forkhead box C1 (*FOXC1*) in cancer, especially in studies of basal-like breast cancer (BLBC). Other studies have followed suit, demonstrating that *FOXC1* is not only a major player in this breast cancer subtype, but also in hepatocellular carcinoma (HCC), endometrial cancer, Hodgkin's lymphoma (HL), and non-Hodgkin's lymphoma (NHL). The *FOXC1* gene encodes a transcription factor that is crucial to mesodermal, neural crest, and ocular development, and mutations found in *FOXC1* have been found to cause dominantly inherited Axenfeld-Rieger Syndrome (ARS). Interestingly, while *FOXC1* missense mutations that are associated with ARS usually reduce gene activity, increased *FOXC1* function now appears to be often linked to more aggressive cancer phenotypes in BLBC, HCC, HL, and NHL. This review discusses not only the role of *FOXC1* in cancer cell progression, proliferation, differentiation, and metastasis, but also the underlying mechanisms of how *FOXC1* can contribute to aggressive cancer phenotypes.

## The FOX family and cancer

In recent years, a number of FOX family members have been linked to tumorigenesis, carcinogenesis, and the survival of malignant cell growth [1, 2]. Members of the FOXA, FOXC, FOXM, FOXO, and FOXP subclasses of FOX proteins, in particular, were found to have direct effects on the initiation, maintenance, progression, and drug resistance of cancers [2]. For example, the removal of *FOXM1*, which is known to play an integral role in G1-S and G2-M cell cycle progression and mitotic spindle integrity [3], results in the inability to commence mitosis in mice [4]. Furthermore, the overexpression of *FOXM1* accelerates the proliferation and progression of prostate cancers in mouse models [5]. The widely studied FOXO proteins are key negative regulators of tumour suppression, as the simultaneous deletion of *FOXO1*, *FOXO3*, and *FOXO4* alleles in somatic cells invokes thymic lymphomas and systemic haemangiomas in mouse models [6]. As such, many FOX family members are desirable new avenues for further research as possible therapeutic targets in cancer treatment. Of these, perhaps the most important is the FOXC1 forkhead transcription factor (TF), which has been shown recently to have profound and critical roles in several disparate cancer types following its initial identification as a key prognostic indicator of basal-like breast cancer (BLBC) [7–9]. Within this paper we will review FOXC1's impact in cancer, focusing on FOXC1's role in signaling pathways, gene regulation, and interactions with other proteins and how these factors affect the nature of this malignant disease.

## The FOX family

**Figure 1:**
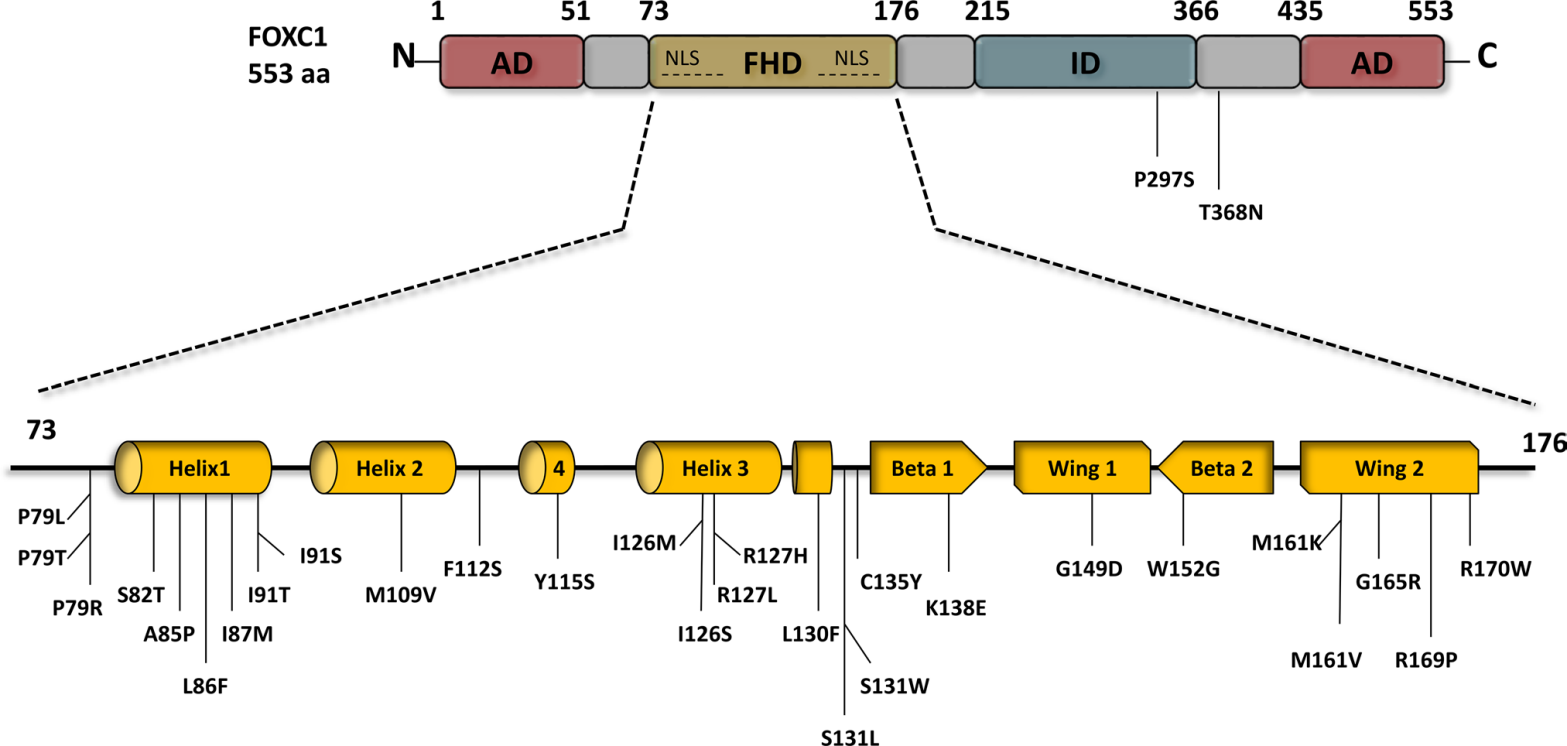
*FOXC1* schematic structure and *FOXC1* missense mutations *FOXC1* protein contains two activation domains (AD) that are located at the N-terminus 1-51 aa, and the C-terminus 435-553 aa, both of which play a main role in *FOXC1* activation. Engineered *FOXC1* proteins that lack either the N- or/and C- terminus have reduce activity and improper functions. *FOXC1* protein localizes to the cell nucleus via two nuclear localization sequences (NLS), and binds to DNA via the forkhead domain (FHD) 73-176 aa. To date 28 point mutations have been identified in the FHD of *FOXC1*, most of which are linked to ocular defects and malformations. Deletion of the inhibitory domain (ID) 435-533 aa.significantly increases *FOXC1* activity. In contrast to the two ADs that activate *FOXC1*, specific residues in the ID experience post-translational phosphorylation and as a result inhibit FOXC1function.

The FOX gene family, otherwise known as the Forkhead box gene family, is a group of highly evolutionarily conserved genes [10] with a common DNA-binding domain of 110 amino acids known as the forkhead box or “winged helix” domain (FHD) (Figure 1) [3, 11]. The general structure of the FHD consists of three α-helices, three β-sheets, and two “wing” regions situated on either side of a third β-sheet – this produces the “butterfly-like” characteristic that inspired the moniker of the “winged helix domain” [10]. FOX transcription factors (TFs) have distinct roles in embryonic and adult development [12], and are connected to chromatin remodeling as well as nuclear delocalization [2, 11, 12]. The orthologue of this functionally diverse family was found nearly three decades ago in *Drosphila melanogaster*, in which a mutation in the homeotic gene *forkhead* (*fkh*) was found to inhibit gene expression and manifest aberrant head structures [13]. Since then, more than fifty different forkhead proteins have been discovered in humans, classified in subgroups ranging from FOXA to FOXS [2, 10, 11].

## FOXC1: background, function, structure, and mutations

*FOXC1*, which is also known as Mf1, Fkh-1 [11] or FREAC3 [14], is a single exon gene located at 6p25 encoding a 533 aa protein that localizes to the nucleus, where it can bind to the DNA and regulate gene expression [15]. *FOXC1* is an essential component of mesodermal [16], neural crest [17] and ocular development [18–20] and is often studied and discussed in relation to Axenfeld Rieger syndrome (ARS). ARS can be caused by *FOXC1* mutations [3, 21] and involves the abnormal development of the anterior segment of the eye. Importantly, 50% of ARS patients go on to develop high ocular pressure [22]. *FOXC1* is also associated with Dandy-Walker malformation, which is a condition in which patients suffer from an underdeveloped cerebellum and enlarged posterior fossa [21, 23]. While this gene is undoubtedly an integral developmental transcription factor – the deletion of both *FOXC1* alleles in mice leads to not only issues in ocular development, but it also gives rise to hydrocephalic, cardiac, organogenesis, and skeletal anomalies, thus increasing the propensity for neonatal mortality [15, 16, 24]. More recently, *FOXC1* has been found to play a role in carcinogenesis and tumorigenesis, most notably in BLBC [25]. *FOXC1*, however, is not only involved in BLBC – studies have shown that *FOXC1* plays a role in the interleukin-8 inflammatory pathway associated with hepatocellular carcinoma [26, 27] while other studies reveal *FOXC1*'s involvement in endometrial cancer progression via miRNA 204 and miRNA 495 [28, 29]. The relationships between FOXC1 and these cancers will be expanded upon later in this review.

Like others of the FOX family, the phosphoprotein FOXC1 [22] possesses the “winged-helix” structure in its DNA binding domain (Figure 1). The third α-helix of the “winged helix” crosses perpendicularly to the DNA helical axis, creating a sequence-specific contact with the major groove in the core base sequence GTAAATAAA-3′ [30–32] to which FOXC1 has a strong affinity, as determined through *in vitro* experiments [14]. There are additional protein-DNA contacts possible in the second wing region [32]. *FOXC1* regulates transcription through its N- and C- terminal activation domains as well as a phosphorylated transcription inhibitory domain [15].

The transactivation of FOXC1 requires the N-terminal activation domain and a glutamine-rich/hydrophobic C-terminal activation domain, which are located at residues 1 – 51 and 435 – 553, respectively (Figure 1) [15, 33]. HeLa cells transfected with the full-length FOXC1 (1-553) cDNA were compared to empty vectors with a luciferase reporter and were found to have a ten-fold induction of luciferase activity compared to the latter [15]. In addition, when the 1-29 or 1-51 N-terminal amino acids were deleted, the luciferase activity decreased 50% to 55% respectively, leading to the proposal that these residues in the N-terminus are essential to the full activation of FOXC1 [15]. A FOXC1 protein expressed lacking both the N- and C-terminal regions yielded similar luciferase levels to the empty vectors [15]. Furthermore, the activity at these domains is mitigated by a phosphorylated inhibitory domain (ID). The phosphorylation of residues in the ID play a role in FOXC1 stability and activity [15]. Berry and his colleagues have shown that the phosphorylation of FOXC1 through the activation of the ERK1/2 mitogen-activated protein kinase (MAPK) pathway is critical in stabilizing FOXC1 in HeLa cells [22]. The trypsin digest pattern of FOXC1 is altered by its phosphorylation, further bolstering the proposal that FOXC1 is regulated through conformational change as altered conformation may affect the availability of protease-protein contact. It was suggested also that FOXC1 may be regulated by kinase and phosphatase activity [32].

**Figure 2:**
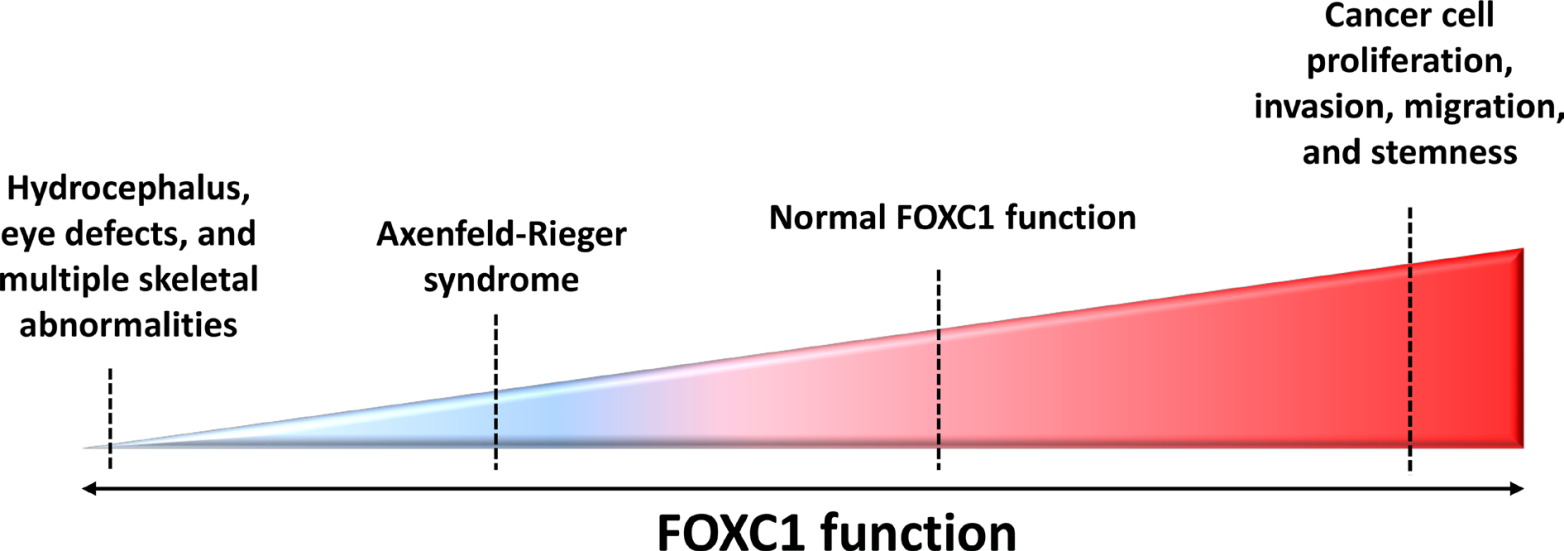
FOXC1 function and activity in human diseases *FOXC1* has been shown to play an integral role in development and adulthood, with both increased and decreased *FOXC1* function linked to abnormal disease phenotypes. For example, due to profound defects in ocular development, hydrocephaly, cardiac organogenesis and skeletal anomalies, homozygous null *Foxc1* mice do not survive past birth [16]. Mutations in *FOXC1* are shown to hinder FOXC1-DNA binding activity, *FOXC1* protein level and stability, as well as *FOXC1* translocation to the nucleus – all of these defects resulting in Axenfeld-Rieger Syndrome (ARS). More recently, *FOXC1* has been demonstrated to have a key role in cancer progression. Contrary to the reduced *FOXC1* function observed in ARS, recent studies are linking escalated *FOXC1* protein levels to the development of more aggressive phenotypes in cancers such as breast cancer, HCC, and endometrial cancer.

*FOXC1* point mutations have been reported and studied [34–41]. These mutations have been shown to reduce FOXC1 protein level, FOXC1 transactivation, and/or FOXC1's DNA binding ability [36, 37, 41]. To date, 31 missense variants in ARS patients have been identified in *FOXC1*, 29 of which occur within the forkhead domain (Figure 1). Normally, FOXC1 is located in the nucleus where it binds to DNA to activate or inactivate other genes. Missense and nonsense mutations within the *FOXC1* forkhead domain that alter FOXC1 translocation to the nucleus reduce its function. For example, Saleem and colleagues functionally characterized various mutations throughout the forkhead domain of FOXC1 (Figure 1). They found that FOXC1 with either the S82T, L86F, F112S, or I126M mutation displayed 80-100% nuclear localization compared to wild-type FOXC1, 61-80% for either P79L, P79T, or S131L, 41-60% for I91T, and 0-20% for either I91S or R127H [33, 37, 41]. These mutations had shown to reduce FOXC1 activity due to impaired FOXC1 translocation to the nucleus. Aside from nuclear translocation, mutations within the FHD of FOXC1 can impair binding activity of FOXC1 to its target genes. Specifically, the R127H and S131L mutations in α-helix3 reduced FOXC1 binding to DNA by 90 % compared to wild-type FOXC1 binding efficiency [33, 37, 42]. Moreover, some mutations in the FHD were reported to cause other molecular defects to FOXC1. In particular, the I87M, R127H, and H128R mutations reduce protein stability, alter binding specificity, and extend protein half-life, respectively [33, 37, 42]. Missense mutations that alter FOXC1 translocation to the nucleus, binding to DNA, and protein stability consequently reduce FOXC1 function. In addition, recently, gain of function mutations have also been found to be rare causes of dominant glaucoma [40]. Similarly, loss and gain of function mutations in FOXC2 have been shown to cause lymphedema-distichiasis [43]. Together these mutations consequences are likely to be responsible for the developmental anomalies in ARS (Figure 2) and lymphedema-distichiasis patients.

Interestingly, the increased function of FOXC1 has also been linked to malignancy. In contrast to how reduced FOXC1 underlies ARS, an increase in function and activity of FOXC1 is responsible for cancer cell proliferation, differentiation, survival and metastasis (Figure 2). The nature of FOXC1's contribution to this malignancy is further discussed in this review.

## FOXC1 and basal-like breast cancer (BLBC)

Currently, out of all the associations *FOXC1* has with different forms of cancer, *FOXC1's* relationship with breast cancer, specifically BLBC, is the most elucidated. BLBC is a form of triple negative breast cancer (TNBC), in which the receptors for estrogen (ER), progesterone (PR), and human epidermal growth factor receptor 2 (HER2), are all not overexpressed [44]. BLBC usually presents with high histologic grade, aggressive clinical features, poor prognosis, and a propensity to metastasize to the brain and lung [45]. Genes normally expressed in basal/myoepithelial cells are also expressed in the normal mammary gland in BLBC, and it must be noted that BLBC prognosis is usually determined with additional immunohistochemistry markers such as basal cytokeratins CK5/6, CK14, CK17, and epidermal growth receptor (EGFR) to increase accuracy [46]. BLBCs have been defined in different studies using differing sets of diagnostic markers. For example, Nielsen et al., 2004 defined BLBC on the basis of negative ER and HER2 expression but positive basal cytokeratin, EGFR, and/or c-kit expression [47], while other groups used the combination of negative ER and HER2 expression and positive CK5, P-cadherin, and p63 expression [48] or positive vimentin, EGFR, and CK5/6 expression [49]. Thus, there is no internationally accepted definition for basal-like cancers, and there is no genetic test available in clinical practice to identify these tumors. Although the gold standard for the diagnosis of BLBC is gene expression profiling, emerging data suggests that FOXC1 is a sensitive biomarker for triple negative breast cancers, and in particular, BLBC [50, 51].

## FOXC1 is a key prognostic indictor for basal-like breast cancer

**Figure 3:**
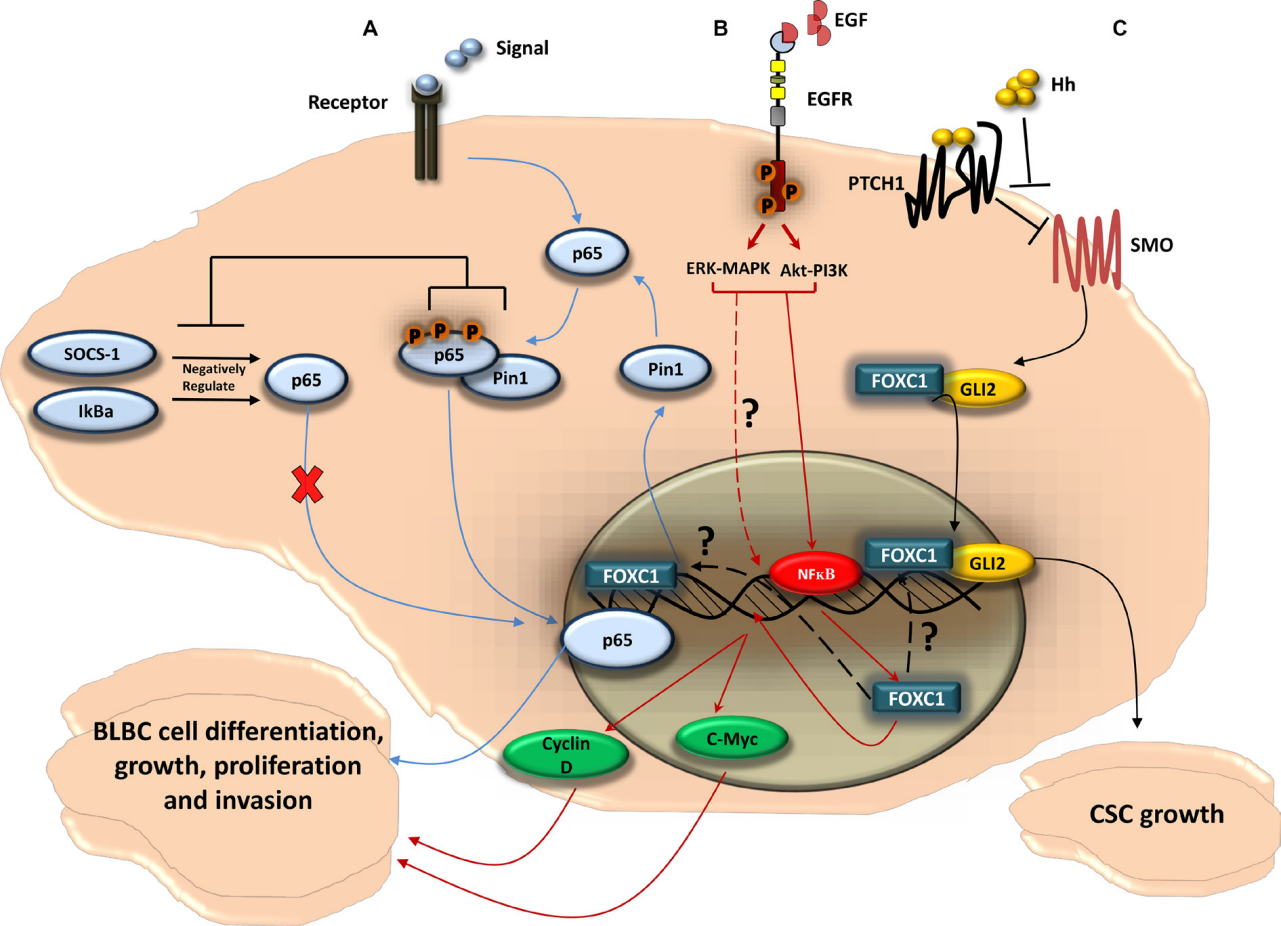
FOXC1-signaling pathways in BLBC (**A**) *FOXC1* regulates the function of the NF-κB pathway in BLBC cell; NF-κB pathway can be activated as a cellular response to stimuli. Once activated, the NF-κB subunit p65 get phosphorylated and translocated to the nucleus where it binds to DNA. The p65 activity is negatively regulated by the ubiquitin ligase cytokine signal inhibitor SOCS-1 [133] that sends p65 to the proteasome for degradation, and by IκBα that plays a role in the steady-state cytoplasmic localization of p65 dimers, thus preventing p65 nuclear localization and DNA binding [134]. The NF-κB pathway activity has been linked to tumorigenesis. In BLBC cell, *FOXC1* regulates the expression of Pin1, a peptidyl-prolyl isomerase, that regulates the activity of p65 [133] and has been linked to tumor development [135]. Pin1 physically binds to p65 in the cytoplasm. This physical binding thus blocks p65 association with SOCS-1 and IκBα, as a result inhibits the p65 degradation. This then leads to p65 phosphorylation and p65 translocation to the nucleus. p65 binds to DNA and activates genes that enhances BLBC cell growth and proliferation. (**B**) EGFR, via MAPK-ERK and PI3K-Akt pathways, upregulates *FOXC1* in BLBC; upon activation of EGFR by the ligand EGF, two of the classical pathways Mitogen-Activated Protein Kinase (MAPK) and Phosphatidylinositol-4,5-bisphosphate 3-kinase (PI3K) can be activated. The PI3K and MAPK pathways thus upregulate *FOXC1* protein and mRNA expression through the ERK and Akt proteins. It has been shown that Akt and ERK phosphorylate and activate NF-κB that leads to its translocation to the nucleus [130]. NF-κB then would bind to *FOXC1* promoter region and increases *FOXC1* transcription activity. *FOXC1* then would enhance the expression of the transcription factor c-Myc and Cyclin D, in which both play a key role in BLBC cell growth, proliferation, and invasion. (**C**) *FOXC1* activates Smoothened-independent Hedgehog Signaling; the ligand Hh binds to the receptor Patched 1 (PTCH1) which allow SMO to activate the transcription factor Glioma-Associated Oncogene Family Zinc Finger 2 (GLI2). *FOXC1* can activate GLI2 independently from SMO, where the *FOXC1* N-terminal domain (aa 1-68) binds directly to a certain internal region of GLI2 (aa 898-1168), increasing GLI2-DNA transcription activity. *FOXC1* activation of the non-canonical Hh signaling can result in cancer stem cell growth and expansion, consequently produces the BLBC stem-like phenotype.

Recently, a central role in BLBC for FOXC1 has been clearly established [7, 8, 25, 50–52]. As indicated in (Figure 3), FOXC1 is associated with BLBC through critical signaling pathways [7, 8, 52] and is directly linked to tumor metastasis and invasion [25].

As a transcription factor of the functionally versatile FOX family, FOXC1 has a role in many gene regulatory pathways [7, 8, 11, 12, 23, 52]. Of these pathways, the most intriguing from the perspective of cancer biology are those involved in cell growth, proliferation, differentiation, invasion, and cancer stem cell growth (Figure 3). FOXC1 is consistently and exclusively over-expressed in BLBC when compared to other breast cancer molecular subtypes in multiple, independent, gene expression microarray datasets [50]. Ray and his colleagues determined a significant positive correlation between high FOXC1 activity and FOXC1 mRNA expression and BLBC [50]. Further expansion on these relationships yielded that brain metastasis-free survival was significantly tied to high FOXC1 mRNA levels. Moreover, the ectopic overexpression of FOXC1 invoked more aggressive breast cancer phenotypes, including epithelial-mesenchymal transition, increased cell proliferation, increased migration, and increased invasion [50]. This association of increased FOXC1 levels with BLBC and poor prognosis appears to be the result of the aggressive cell phenotypes that result from over-expression of FOXC1 [50, 51]. Knockdown of FOXC1 expression by siRNA in BLBC cell lines significantly decreased cell proliferation, migration, and invasion [50]. Furthermore, several studies have reported on the interaction between FOXC1 and signaling pathways. For example, FOXC1 can regulate the BLBC cells by activating the NF-ĸB signaling pathway (Figure 3) [52]. FOXC1 also mediates the function of EGFR [8], which has previously been suggested as a surrogate biomarker in BLBC [46]. While the activation of EGFR leads to the upregulation of FOXC1 expression through ERK- and AKT, FOXC1 is a necessary component in EGF-invoked cell proliferation, migration, and invasion (Figure 3) [8]. More recently, Han et al., 2015 found that FOXC1 interacts with Gli2 in different BLBC cell lines through direct binding, and that FOXC1 mediates the non-canonical Smoothened (SMO)-Independent Hedgehog (Hh) signaling that establishes the BLBC stem-like phenotype and anti-Hh sensitivity (Figure 3) [7]. These findings clearly suggest that FOXC1 is a specific biomarker for BLBC. Since FOXC1 has a critical role in the aggressive BLBC cellular phenotype, modulation of FOXC1 activity could lead towards effective BLBC treatment.

## Hepatocellular carcinomas and FOXC1

FOXC1 has recently also been shown to have key roles in other cancers as well. Hepatocellular carcinoma (HCC) is a subset of liver cancer [53], and is ranked as the fifth most common cancerous cause of death in men and the eighth most common cancerous cause of death in women [54]. HCC comprises 85% to 90% of all primary liver cancers [53]. The most common risk factor for HCC is cirrhosis, chronic deterioration of the liver with inflammation, cell degeneration, and fibrous thickening of tissue, as well as possibly jaundice, palmar erythema, and gynecomastia [55]. Other major risk factors include chronic consumption of alcoholic beverages, hepatitis B, hepatitis C, and non-alcoholic fatty liver disease [53, 55], while factors such as Wilson's disease, hereditary hemochromatosis, alpha1-antitrypsin deficiency, primary biliary cirrhosis, and autoimmune hepatitis are less prevalent [56]. Methods of treatment include staging-guided treatment, surgical resection, liver transplantation, local ablation (especially radiofrequency ablation), trans-arterial chemoembolization and radio-embolization, and targeted molecular therapy [57].

In the past few years, *FOXC1* has emerged as a transcription factor with a potentially crucial role in the metastasis of HCC [26, 27, 58]. Microvascular invasion (MIV) has been singled out as one of the most crucial clinicopathological risk factors to predict the carcinoma's propensity for metastasis [59] and early recurrence despite curative liver resection and orthotopic liver transplantation [59, 60]. The epithelial-mesenchymal transition (EMT) is a process where polarized epithelial cells can acquire mesenchymal attributes such as fibroblastoid morphology, characteristic gene expression changes, increasing potential for motility, and increased invasion and metastasis in cancer [61], and is implicated in the MIV formation process [58]. Xu et al. discovered that the suppression of *FOXC1* expression reverses the EMT process, as evidenced by the decreased expression of mesenchymal markers Vimentin and N-cadherin, the decreased translocation of β-catenin to the nucleus, and the increased expression of epithelial markers ZO-1 and Claudin-1 in response to *FOXC1* knockdown [58]. Furthermore, the expression of FOXC1 was found to be elevated after the eighth day of a 14-day treatment of Huh7 (non-metastatic HCC cell line) cells with TGF-β1 and found that *FOXC1* knockdown has no effect on TGF-β isomer expression [58], providing evidence that *FOXC1* may operate downstream from TGF-β1. TGF-β1 is a multifunctional cytokine [62] that inhibits cell growth by arresting cells in any portion of the G0/G1 phase through various mechanisms, for example, through the suppression of retinoblastoma protein (Rb) phosphorylation by upregulating specific CDK inhibitors (i.e. P27/KIP1, P15/INK4B, and P21/CIP1) [62–64]. From the study of *FOXC1*'s interactions with other TFs involved in EMT regulation, *FOXC1* is surmised to also operate downstream from other EMT regulators – such as snail, slug, or twist – and to help invoke the mesenchymal portion of the EMT process [58]. Increased N-cadherin mediation of heterotypic contacts between endothelial and melanoma cells as well as increased β-catenin translocation to the nucleus found in trans-endothelial migration [65] supports the argument that overexpressed *FOXC1* favours MIV generation [58].

In a similar study conducted by Xia et al, upregulated levels of *FOXC1* in HCC tissues were linked to poor prognosis in HCC patients [26]. *FOXC1*'s role in inducing the EMT process to increase cancer cell migration and invasion offers a possible explanation for how overexpressed *FOXC1* mRNA was found to elevate HCC metastatic potential *in vitro* and encourage lung cell metastasis *in vivo* [26]. The inhibition of the cell adhesion mediator E-cadherin by *FOXC1* transactivation of E-cadherin's direct repressor, *Snai1*, as well as the overexpression *FOXC1's* direct transcriptional target, NEDD9, have been positively correlated with increased cancer cell migration and invasion [26, 27] and may be involved in the *FOXC1* regulation of EMT. As an inflammation-induced cancer, poor HCC prognosis may also be exacerbated via its inflammation signalling pathways. The pro-inflammatory CXC cytokine interleukin 8 (*IL-8*), secreted by tumour cells and tumour-associated macrophages (TAMs), are critical factors that bind to the receptors *CXCR1* and *CXCR2* to promote tumour angiogenesis and metastasis [66]. HIF1-α (hypoxia-inducible factor 1 alpha) binding sites are key factors in the IL-8 signaling pathway that are associated with the α-subunits [66–68] of the larger HIF1 αβ-heterodimeric DNA binding factor, which mediates hypoxia-inducible activity on the 3′ enhancer of erythropoietin [66, 69, 70]. HIF1-α is often overexpressed in cancer [67, 71, 72], and interactions between HIF1-α and the vascular endothelial growth factor (VEGF) were found to perform a role in angiogenesis [73–76]. VEGF is a known gene target for FOXC1, mainly operating in blood vessel maturation and lymph vessel sprouting [3, 77].

In HCC specifically, a mutation in the HIF1-α binding sites in the sequence between nt-960 and -635 in the *FOXC1* promoter region of HCC cells leads to reduced *FOXC1* promoter activity due to decreased *IL8*-mediated binding of HIF1-α to the *FOXC1* promoter [27]. Out of four kinase inhibitors – the inhibitors of phosphatidylinositol-3-kinase (PI3K), extracellular signal-regulated kinase (ERK), c-Jun-N-terminal kinase (JNK), and p38 – only the PI3K inhibitor made significant changes to *IL-8*-induced *FOXC1* expression by inhibiting HIF-α binding to the *FOXC1* promoter region [27]. *IL-8* is therefore likely to regulate *FOXC1* through the PI3K/Akt/HIF-α signalling pathway in HCC [27, 58]. The transactivation of genes *CXCR2* and *CCL2* – which are correlated with tumour angiogenesis and metastasis as well as macrophage infiltration and breast metastasis promotion respectively [27, 78] – with upregulated *FOXC1* also plays a role in inflammation-based HCC metastasis [27], indicating that there are many pathways through which *FOXC1* influences HCC metastatic potential.

Therefore, overexpressed *FOXC1* was found to not only aggravate the malignant development of HCC by favouring the EMT and MIV generation [26, 27, 57, 58], but also transactivate genes related to angiogenesis and metastasis, *CXCR2* and *CCL2*, through the *IL-8*-regulated PI3K/Akt/HIF-α inflammatory signalling pathway. [27, 66, 78] Advances in the understanding of the underlying mechanisms involved in the relationships between high *FOXC1* expression and increased HCC metastatic potential may yield effective targets for precise medical treatment for not only HCC, but other cancers as well.

## Endometrial cancer and FOXC1

Endometrial cancer, a subset of uterine cancer, is not only the third most prevalent gynaecologic malignancy worldwide, but also the most common cancer pertaining to the female genital tract [79]. All tumours from the body of the uterus to the cervix, but not the adenocarcinomas of the endocervix spreading up to the body, are included in this definition [80]. There are two subsets of endometrial carcinomas: while Type I tumours are comprised mainly of endometrioid adenocarcinomas whose development is associated with estrogen hyperplasia and express steroid hormone receptors, Type II tumours, the mostly serous and clear-cell carcinomas, are usually negatively or weakly positive for steroid hormone receptors, poorly differentiated, and of a high grade [81]. The development of endometrial cancer is associated with increased coding errors and somatic mutations, thought to be caused by extensive endometrial cell proliferation from long-term exposure to estrogen [81, 82]. Other factors have also been associated with elevating the risk for endometrial cancer, such as early menstruation, late menopause, infertility, nulliparity, obesity, and estrogen replacement therapy without involvement of a progestin [82].

Although the studies of *FOXC1* in basal-like breast cancer and hepatocellular carcinoma are well underway, the investigation of *FOXC1* in endometrial cancer has just begun. Studies in recent years have shed light onto the FOX transcription factor's role in a variety of pathways that are involved in endometrial tumorigenesis, especially focusing on the oncogenic role of *FOXC1* in pathways involving microRNAs (miRNA) [28, 29].

In 2007, Wong et al. reported *FOXC1* as a newly found differentially regulated gene with a 5.21 fold upregulation in endometrioid endometrial cancer [83]. MicroRNAs – in particular, miRNA 204 (miR204) and miRNA 495 (miR495) – appear to play a part in bridging the observed relationship between *FOXC1* and endometrial cancer [28, 29]. HEC1A and Ishikawa endometrial cancer cell lines treated with pre-miR-204 yielded minimized levels of *FOXC1* protein and subsequently, reduced cell migration [28]. Through luciferase reporter assays, Chung et al. also demonstrated that the miR204 regulates *FOXC1* expression by interacting with binding sites on the *FOXC1* 3′UTR (3′ untranslated region) [28]. Further studies by Chung et al. suggest that there may be a potential downstream pathway regulated by miR204 responsible for triggering endometrial cancer progression [28].

On the other hand, miR495 was initially demonstrated to be involved in a breast cancer stem cell pathway where it is activated by the transcription factors E12/E47 and down-regulates E-cadherin and *REDD1* to promote oncogenesis and hypoxia [84]. Li et al. provide evidence that miR495 also plays a role in the inhibition of gastric cancer cell migration through direct interactions with a member of the PTP (protein tyrosine phosphatase) family, PRL-3 [85, 86]. In endometrial cancer, miR495 takes on the same inhibitory role as it does in gastric cancer; the miRNA suppresses cancer cell growth via cell apoptosis and was shown to inhibit migratory abilities *in vitro* with Matrigel-lacking transwell assays [29]. The miR495 binds to sites 667 and 1629 of the 3′ UTR region of *FOXC1* and negatively regulates the endogenous expression of the FOX family member at the post-transcriptional level [29]. Further experiments *in vivo* asserted that miR495 suppressed carcinogenesis while downregulating *FOXC1* [29]. Intriguingly, a rescue experiment involving the overexpression of *FOXC1* abrogated miR495's inhibition of cell growth and migration as well as promotion of apoptosis in AN3CA and KLE cells (endometrial cancer cells) [29]. These findings provide a strong argument for *FOXC1*'s role as a target of miR495 in the miR495-regulated malignancy phenotype found in endometrial cancer.

Thus far, *FOXC1* appears primarily to be a potential oncogene in not only hepatocellular carcinoma, but in endometrial cancer as well. While MIV generation and *IL-8*-regulated PI3K/Akt/HIF-α inflammatory signalling pathway are the focus of *FOXC1* regulation in HCC [26, 27, 57, 58], microRNAs take spotlight in *FOXC1*'s relationship with endometrial cancer. In particular, the upregulation of miRNA 204 and miRNA 495 was shown to invoke tumour suppression through decreased *FOXC1* protein expression [28, 29]. Further research illuminating the pathways in which the miRNAs and *FOXC1* interact in endometrial cancer will allow for an understanding of how to halt endometrial cancer progression and suppress endometrial cancer cell migration. With roles found in the development of other cancers such as breast cancer and gastric cancer, miR495 is also an viable avenue for deeper investigation – the existence of a common oncogenic pathway would be crucial to the development of a generalized but effective treatment plan that will be able to counteract a variety of cancers.

## Lymphoma (hodgkin's and non-hodgkin's) and FOXC1

There are two main classifications of lymphoma: Hodgkin's and Non-Hodgkin's [87–90]. Hodgkin's lymphoma (HL) can be further defined as nodular sclerosis, mixed cellularity, lymphocyte-rich, lymphocyte-depleted, and nodular lymphocyte predominant HL(NLPHL) – the first four together comprise what is known as ”classical HL” [89, 91, 92]. The organs implicated in HL include the peripheral lymph nodes, and sometimes the liver, the lungs, and bone marrow [89]. Conversely, Non-Hodgkin's lymphomas (NHL) are a diverse group of lymphoproliferative disorders stemming from B-, T-, or natural killer (NK) lymphocytes [90]. In a pooled analysis of eight case-control studies of NHL, single nucleotide polymorphisms (SNPs) in tumour necrosis factor (*TNF*) and interleukin-10 (*IL-10*) genes –which were responsible for encoding key cytokines in inflammatory response and immune balance – were associated with a risk of NHL, particularly in diffuse large B-cell lymphoma [93, 94]. Patients with autoimmune diseases such as rheumatoid arthritis (RA), celiac disease, systemic lupus erythematosus (SLE) and Sjögren's syndrome were also associated with a higher risk of NHL [90, 95].

The overexpression of *FOXC1* has been consistently observed in the occurrence and development of Hodgkin's lymphoma [96, 97]. For example, in the HL cell lines KM-H2 and U-HO1, Nagel and colleagues have identified elevated levels of *FOXC1*, suspected to be caused by chromosomal aberrations at 6p25 [97]. Further experiments support the possibility that *FOXC1* directly regulates *MSX1*, a NKL homeobox gene downregulated during B-cell development [98] and overexpressed in cell lines derived from mantle cell lymphoma and acute myeloid leukemia [96]. A site found upstream of *MSX1* at - 2661bp that is identical to the *FOXC1-*binding site found in the closely related *MSX2* gene, predicts the direct binding of *MSX1* by *FOXC1* [96, 98].

*ZHX2* is a B-cell specific factor that plays a role in differentiation and apoptosis [97, 99], where through expression analyses, it was found in hematopoietic cell lines and primary cells that *ZHX2* acts as a tumour suppressor for HL and multiple myeloma [100]. Studies show that *ZHX2* may have an influence over the NOTCH pathway [101], a pathway often active in HL and mediates apoptosis in a variety of B-cell related malignancies [102], as deduced from its activation of NOTCH-target genes *HES4* and *HOXA*5 [100, 102, 103]. The *FOXC1-*provoked deregulation of *MSX1* and shuttle-protein encoding gene, *IPO7*, is implicated in the downstream inhibition of *ZHX2* – specifically, *MSX1* and its co-repressor histone H1C inhibit *ZHX2* expression while *IPO7* encodes for a shuttle protein that transports histone H1 proteins into the nucleus and overexpression of these genes results in decreased *ZHX2* levels [96, 97, 104, 105]. Therefore, aberrations in the chromosomal structure of *FOXC1* produces abnormalities in *MSX1* and *IPO7* regulation, which in turn renders *ZHX2* incapable of suppressing HL tumorigenesis.

While investigations into *FOXC1's* connection to HL centered around *MSX1, IPO7*, and *ZHX2* [96, 97, 104, 105], new studies of *FOXC1* in NHL revolve around the activating protein (AP-1) Jun protein family. Jun proteins can exist as either homo- or heterodimers [106] that are usually activated in response to stress signals such as UV irradiation. Jun proteins also promote mitogen-induced cell cycle progression in growth factor pathways, or regulate apoptosis through the modulation of cancer suppressor p53 protein and cyclin D1 expression [107]. DLBCL (diffuse large B-cell lymphoma) is a common aggressive manifestation of non-Hodgkin's lymphoma that has at least 3 molecular subtypes with distinct prognoses, each differing in the expression of hundreds of genes [108]. The knockdown of the genes encoding c-Jun and JunB in DLBCL cells results in an inability to produce the factors of *IL-6* and *IL-10*, causing growth inhibition in neoplastic cells, especially in NHL [108–110]. Through gene expression profiling studies of cells with down-regulated c-Jun and JunB expression, the genes coding for matrix metalloproteinase 7, adhesion molecule CD44, vitronectin receptor (*ITGAV*), fractalkine receptor (*CX3CRI*), and most notably, *FOXC1* – all known to encourage the metastasis and invasion of solid tumours [50, 111–118] – correspondingly displayed decreased expression [119]. Thus elevated Jun protein levels are linked to the increased migration and invasion of solid tumours in NHL through *FOXC1* expression [119].

The activated B-cell-like subtype (ABC-DLBCL) is associated with the poorest prognosis, which is linked with the constitutive activation of the NF-κB pathway [119, 120]. The scaffold molecule CARD11, which is exclusively expressed in hematopoietic cells [121, 122] and plays a well-known role in antigen-induced NF-κB signaling activation [123–127], is correlated with the signaling induction of c-Jun and JunB in T cells. Elevated CARD11 activity drives the activation of c-Jun and JunB in DLBCL [118, 128] – the constant activation of CARD11 leads to decreased ubiquitination and degradation of c-Jun in human DLBCL cell lines, suggesting that CARD11 is responsible for the stabilization and accumulation of c-Jun [119]. Along with elevated JunB protein levels, the elevated c-Jun levels result in ectopic AP-1 activity that promotes lymphoma interaction with the microenvironment as well as lymphoma dissemination into extra-nodal sites such as the bone marrow *in vivo* [119], rendering more aggressive lymphoma conditions. Additional investigations examining the role of *FOXC1* in the CARD11-Jun pathway are necessary to determine if *FOXC1* plays a role in augmenting poor prognosis in DLBCL.

Although the pathways through which *FOXC1* influences Hodgkin's and non-Hodgkin's lymphoma are different, there is one commonality that is observed: the overexpression of *FOXC1* contributes to the further development of the lymphomas through either differentiation and apoptosis or migration and invasion [96, 97, 117]. In HL, *FOXC1* is proposed to regulate the NKL homeobox gene involved in B-cell development, *MSX1*, which in conjunction with the shuttle-protein encoding gene *IPO7*, inhibits the B-cell specific factor involved in differentiation, apoptosis and the NOTCH signalling pathway, *ZHX2* [96–98, 100, 104]. On the other hand, in NHL, the focus is placed on the relationship between *FOXC1* and the CARD11-Jun pathway, where elevated Jun protein levels were linked to elevated *FOXC1* levels, which in turn is linked to the increased occurrence of migration and invasion of solid tumours in NHL [118, 128]. However, the commonality observed is not only limited to HL and NHL. In all the cancers discussed thus far, the ectopic overexpression of *FOXC1* is always linked to increased aggression in cancer disease phenotypes, indicating *FOXC1*'s potential as a major oncogene. The elucidation of existing *FOXC1*-related cancer pathways as well as the investigation into the role of *FOXC1* in other cancers may yield not only a strong general prognostic biomarker for belligerent cancer phenotypes, but also precise genetic treatments for individual cases of malignancy.

## CONCLUSIONS

*FOXC1* is a master regulator of gene expression that plays a critical role in embryonic development, consistent with the fact that FOXC1 mutations are associated with developmental anomalies [15, 16, 129] (Figures 1 and 2). More recently, however, studies have linked *FOXC1* activity to the aggressive phenotype in cancer cells. FOXC1 enhances cell invasion, proliferation, metastasis, EMT, and migration in BLBC [25]. however, the cross-talk between these pathways and the underlining mechanisms for their compensation still needs to be elucidated (Figure 3). Although the EGFR-MAPK-PI3K pathway upregulates the expression, activity, and protein level of *FOXC1* [8] (Figure 3), the how and why of *FOXC1* being exclusively expressed in BLBC rather than in other breast-cancer molecular subtypes has yet to be answered. Very recently, Chung and colleagues [130] have shown that NF-ĸB binds to the promoter region of FOXC1 once EGFR is activated by EGF. NF-ĸB binding to FOXC1 can increase FOXC1 transcription activity (Figure 3). It would be interesting to know if FOXC2 [131] is also involved in this cancer circuit. The factors that bind to and regulate FOXC1, for example in response to EGFR-MAPK-PI3K pathway activation, are still being discovered (Figure 3). Recently, FOXC1 has been shown to activate GLI2 in a SMO independent SHH pathway (Figure 3), which partly explains the aggressiveness of BLBC cell and adds a new role for FOXC1 in cancer cell stemness [7]. Moreover, EMT, which plays a key role in the generation and maintenance of cancer stem cells [132] was proposed to be activated by FOXC1 in breast cancer [25] which might explain part of this role of FOXC1 in cancer cell stemness.

In hepatocellular carcinoma, endometrial cancer, as well as both Hodgkin's and Non-Hodgkin's lymphoma, research support the role of *FOXC1* as an oncogene, where upregulated *FOXC1* expression is linked to increasingly aggressive disease phenotypes. *FOXC1* has been implicated in numerous pathways that help determine the nature of different cancers, but the oncogenic mechanisms with which *FOXC1* involved have yet to be completely elucidated. In HCC, increased *FOXC1* expression was shown to encourage cell migration and invasion through its regulation of the EMT and MIV processes, hence elevating cell metastatic potential [58]. *FOXC1* is also associated with the *IL-8* signaling pathway [27] and the transactivation of genes responsible for tumour angiogenesis and metastasis, *CXCR2* and *CCL2* [27, 78]. For HCC, future studies on functionally characterizing factors that work with *FOXC1* in EMT regulation will lead to a better understanding of how EMT and MIV contributes to HCC proliferation. Further investigation of how *IL-8* regulates *FOXC1* through the PI3K/Akt/HIF-α signaling pathway will also improve understanding of HCC pathology.

In endometrial cancer, the downregulation of *FOXC1* by miRNA – specifically miRNA 204 and miRNA 495 – was revealed to inhibit cancer cell growth and migration while increasing the frequency of apoptosis [28, 29]. While currently, the mechanism by which miR204 interacts with *FOXC1* is unclear [28], evidence suggests that miR495 interacts with *FOXC1* through binding on the *FOXC1* 3′UTR [29]. Further exploration into the downstream regulation of *FOXC1* by miR204 and miR495 as well as the pathways in which interactions are involved will lead to a greater understanding of how to mitigate more aggressive phenotypes of endometrial cancer with high metastatic potential. The regulatory role of microRNA 495 should be examined not only in depth in endometrial cancer, but laterally across other cancers as well; determining if the miRNA's interaction with *FOXC1* to mediate cell growth, migration and apoptosis is cancer-specific or common across a variety of cancers would be fruitful.

The overexpression of *FOXC1* found in Hodgkin's lymphoma was linked to abnormalities in the *MSX1* and *IPO7* regulation of *ZHX2*, a gene responsible for tumour suppression; elevated *FOXC1* levels interfere with *ZHX2* moderation of B-cell differentiation and apoptosis, leading to highly aberrant cell growth that may exacerbate HL lethality [96–98]. The *FOXC1, MSX1*, *IPO7*, and *ZHX2* regulatory pathway of HL should thus be further explored; subsequent experiments that determine the molecular mechanisms through which *FOXC1* dysregulates *ZHX2* as well as other components that operate within the pathway would help develop specific methods to hinder increased HL aggression. On the other hand, research into *FOXC1*'s role in non-Hodgkin's lymphoma centers around its interaction with Jun proteins in DLBCL, which play a role in lymphoma interaction with the microenvironment and dissemination into extra-nodal sites [119]. However, this research is still in the early stages, and although *CARD11* and antigen-induced NF-κB signaling activation have been implicated in the regulation of Jun proteins in DLBCL [119], a clear picture of how each component is related to each other and what role *FOXC1* plays has yet to be discerned.

In summary, recent investigations of FOXC1 are beginning to reveal a key protein at the juxtaposition of critical oncogenetic pathways for many cancers. Additional investigations of FOXC1 are likely to not only illuminate the regulation of key pathways in many different cancers, but may identify novel common entry points for treatments of these cancers.
